# An Extraosseous Plasmacytoma of the Nasopharynx

**DOI:** 10.4021/jocmr2009.05.1240

**Published:** 2009-06-21

**Authors:** Ravinder Singh Natt, Gerry O’Sullivan

**Affiliations:** a Wirral University Teaching Hospitals NHS Trust, Arrowe Park Road, Merseyside CH49 5PE, UK

## Abstract

A 75-year-old long-term male smoker and poorly controlled hypertensive presented with a 3-month history of intermittent epistaxis refractory to cauterisation with silver nitrate. Nasopharyngeal examination revealed a mass in the post nasal space. An urgent endoscopic biopsy confirmed an extraosseous plasmacytoma. Post operative radiotherapy was scheduled. No evidence of recurrence of disease following completion of treatment has been detected during clinical surveillance.

**Keywords:**

Epistaxis; Nasopharynx; Biopsy; Plasmacytoma; Radiotherapy

A 75-year-old long-term male smoker and poorly controlled hypertensive presented with a 3-month history of intermittent epistaxis refractory to cauterisation with silver nitrate. Nasopharyngeal examination revealed a smooth mass extending inferiorly from the right fossa of Rossenmuller and effacing the posterior pharyngeal wall (Fig 1). An urgent endoscopic examination and excisional biopsy was scheduled. Haematoxylin and eosin staining confirmed a dense infiltration of plasmacytoid cells. Immunocytochemistry confirmed malignant plasma cells consistent with an extraosseous plasmacytoma.

**Figure 1:**
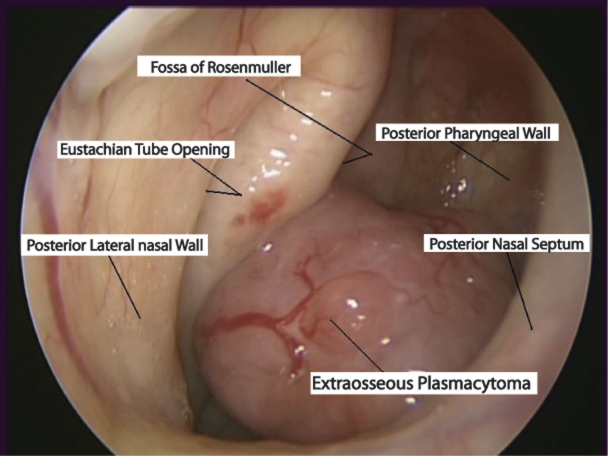
Location of the extraosseous plasmacytoma

Serum and urine assays for Bence Jones protein were negative and there was no evidence of gammopathy. A bone marrow biopsy and Computed Tomography and Magnetic Resonance imaging did not demonstrate any metastasis or skeletal involvement. The Excisional biopsy had been complete and the patient underwent radical radiotherapy with a 45Gy dose in 20 fractions of the nasopharyngeal field.

No evidence of recurrence of disease following completion of treatment has been detected during clinical surveillance.

A plasmacytoma is a very rare discrete solitary mass of neoplastic monoclonal plasma cells, first described by Schridde in 1905 [1]. They are classified into one of two categories; soft tissue and skeletal origin. Extramedullary plasmacytomas represent 3% of plasma cell neoplasms and commonly (80%) originate in the head and neck region [2]. They represent approximately 4% of nasal cavity tumours. There is a greater male preponderance and they occur during the fifth and seventh decades of life [3]. The aetiology remains unknown but viral pathogenesis and chronic irritation from inhaled irritants have been suggested [4].

Tissue biopsy, serum electrophoresis (to exclude myeloma) and radiological skeletal survey with bone marrow biopsy to determine skeletal involvement is necessary for diagnosis. Treatment includes a combination of surgical excision and radiotherapy. Follow-up radiological and electrophoresis assessment is required after treatment to detect recurrences and progression to myeloma (10-30% frequency). The overall 10 year survival is 70% [5].
